# Serving Rural Veterans with Disabilities: A National Survey of Centers for Independent Living

**DOI:** 10.1007/s10900-020-00941-6

**Published:** 2020-11-06

**Authors:** Jennifer Hale-Gallardo, Consuelo M. Kreider, Yuxin Ni, Luz M. Semeah, Zaccheus J. Ahonle, Diane C. Cowper-Ripley, Sharon Mburu, Anthony T. Delisle, Huanguang Jia

**Affiliations:** 1 Research Service, North Florida/South Georgia Veterans Healthcare System, 1601 SW Archer Road (151), Gainesville, FL 32608 USA; 2 Department of Occupational Therapy, University of Florida, Gainesville, FL USA; 3 Veterans Rural Health Resource Center-Gainesville (VRHRC-GNV), Office of Rural Health, Veterans Health Administration, Gainesville, FL USA; 4 Center for Independent Living of North Central Florida, Gainesville, FL USA

**Keywords:** Community integration, Health resources, Veterans health, Psychosocial support systems, Rural populations

## Abstract

This study comprises a systematic national examination of how Centers for Independent Living can and do support Veteran consumers, especially those living in rural communities. This research provides contextualized understanding of rural Veteran needs for community-based services and resources available through Centers for Independent Living. A survey was administered to the leadership of 383 Centers for Independent Living throughout the United States, the majority of which have rural catchment areas and serve rural Veterans through both main and satellite offices. Descriptive univariate analysis was used to describe responses. Study respondents represented a total of 39 states, with 20% of respondents reporting that their consumers were 100% rural and only 3% entirely urban. Services and supports from Centers for Independent Living provided to rural Veterans most frequently included housing, transportation, and peer support. Approximately half of all Centers for Independent Living reported tracking the status of their Veteran consumers.

Veterans with disabilities have unique and complex needs. Veterans can experience a range of health problems related to military service, such as traumatic brain injury [1], spinal cord injury [2], hearing impairments [3], pain [4], and multi-symptom conditions [5]. These conditions often affect mental and social well-being and are associated with homelessness [6], poor health status [7], health-risk behaviors [4], and suicide [8]. Approximately 1 million patients within the healthcare system of the United States (US) Department of Veterans Affairs (VA) affected by a service-connected disability (SCD) live in a rural or highly rural area [9]. This does not include Veterans with non-SCDs; nor does it include Veterans with impairments due to aging, which brings another unique set of concerns [10]. Rural Veterans with disabling conditions experience greater health disparities and more barriers than urban Veterans, including insufficient transportation and social services and lower rates of private insurance and access to specialty care, including mental health services [11, 12].

Veterans affected by disabilities need a range of supports, services and skills to optimize functioning and live independently in their communities [13]. Independent living (IL) refers to the ability to perform self-care and achieve self-determination in the least restrictive environment possible [14]. While the VA offers a broad range of programs and services, rural Veterans may not live close enough to a VA facility to access many services [15] and not every Veteran is enrolled in VA healthcare [16]. In addition, there is increasing recognition that growing numbers of aging Veterans and Veterans with complex conditions may soon exceed the capacity of rural long-term care services [17]. Consequently in recent years, leveraging community-based resources has become a priority for the Veterans Health Administration (VHA) [18].

Key IL resources in the US are Centers for Independent Living (CILs). CILs are community-based, cross-disability, nonresidential, non-profit agencies that emerged in response to previous medical models, which had contributed to experiences of stigma and inequality for individuals with disabilities [19]. Counter to being seen as patients, CILs have historically referred to the individuals with disabilities whom they serve as “consumers.” The IL model is run by people with disabilities for people with disabilities; it emphasizes empowering their consumers with greater choice and control [20]. CILs are congressionally mandated to provide a broad range of services and programs to maximize self-sufficiency, independence, and community integration [21, 22]. Approximately 400 CILs serve urban, rural, and tribal populations; approximately one-third of CILs have rural satellites and many serve rural catchment areas. All CILs offer 5 core services: (1) information and referral, (2) advocacy, (3) peer support, (4) IL skills training, and (5) transition assistance from nursing homes and other institutions to community life.

For rural Veterans living with chronic conditions, CILs are well-positioned to help them avoid institutional care by enhancing their independence and improving long-term outcomes, particularly those with limited access to the VA [23]. While CILs have served Veterans for decades and some of the earliest CIL proponents included Veterans [24], data are lacking on the kinds of support and services provided specifically to Veterans due to the fact that CILs serve all individuals in the community regardless of military status. The purpose of this study was to understand which CIL services are most needed by rural Veterans with disabilities; what services and supports CILs provide to Veterans; and how CILs, VA providers, and policy makers can work together to deliver services needed by rural Veterans.

## Methods

A survey of nationally representative CIL directors was conducted with the approval of the University of Florida Institutional Review Board (IRB).

## Sample and Recruitment

From a comprehensive database of 687 CILs (*n* = 383 main centers; *n* = 298 satellites) across the U.S., 383 CIL directors, each representing a unique CIL main center, were invited by email in April 2019 to participate in the study and report data from fiscal year (FY) 2018. Two reminder invitations were emailed to potential participants in the first week of May and again in the first week of June 2019. The email contained a link to the survey, and survey responses were monitored and tallied one week after each e-mail was sent.

## Survey Instrument

The questionnaire was developed for this study by VA health services and rehabilitation researchers and was vetted by key stakeholders and content experts in the field of IL. Questions and response format (Table 1) were selected and refined based on consensus and were checked to ensure they asked what they were meant to ask, asked for answers within only the dimension of interest, used wording/phrasing suitable to participants, did not include emotionally loaded or vaguely defined phrasing (e.g., double-barreled questions), and were unbiased and not leading; and, when possible, response choices allowed for multiple responses to accommodate a range of possible answers.

**Table 1:** Survey questions sent to 383 Centers for Independent Living (CILs) directors

Domain: survey item	Item response choices
CIL profile	
In what state is your Center for Independent Living (CIL) located?	Drop down menu listing each state in the United States
How many staff members does your CIL have? (Include part-time, full-time, and all funding sources)	0–5; 6–15; 16–25; ≥ 26
Approximately what was the operating budget of your CIL in Fiscal Year (FY) 2018?	Open text response
How many consumers did your CIL serve in FY 2018 (consider all programs regardless of funding)?	Open text response
Of all consumers your CIL served in FY 2018, approximately what percentage were rural?	Open text response
How many Veteran consumers did your CIL serve in FY 2018? (Veterans are defined as any person who has served in the armed forces.) If not tracking Veterans, please mark with an “X”?	Open text response; X = Not tracking Veterans
Veteran Consumers and CIL services for Veterans	
Please drag and drop the following services to rank them in the order of their frequency as used by Veteran consumers at your CIL. (1 being the most used by Veteran consumers; 5 being the least used by Veteran consumers)	Information & referral; Peer support; Advocacy; Independent living skills; Transition from institutions (e.g., nursing homes, correctional facilities)
What other services are most often used by Veterans at your CIL? (Check all that apply)	Transportation; Benefits Assistance; Customer-Directed Personal Assistance; Housing; Community Integration; Work Incentives Planning and Assistance (WIPA); Low Vision/Blind; Deaf/hard of hearing; Employment readiness or other employment services; Emergency preparation/management; Referral for mental health issues/Mental health services; Interpreter service; Durable medical equipment and assistive technology; Other (please specify); Not working with Veterans
Please specify which type(s) of transportation services are most often used by Veterans at your CIL? (Check all that apply)	Volunteer driver programs; Paratransit service; Door-through-door (escort) service; Public transit/fixed route service; mobility training/travel training; Taxi service; Transportation vouchers programs
Please specify which type(s) of benefits assistance services are most often used by Veteran consumers at your CIL? (Check all that apply)	VA Care; Medicare; Medicaid; TRI-Care; Social Security benefits
Please specify which type(s) of housing service are most often used by Veteran consumers at your CIL? (Check all that apply)	Home modification; Accessible housing
Please specify which type(s) of referral for mental health issues/mental health services are most often used by Veteran consumers at your CIL? (Check all that apply)	Post traumatic stress disorder; Traumatic brain injury; Depression/anxiety/suicidal ideation; Smoking cessation; Substance abuse
What are the greatest needs of your Veteran consumers from rural areas?	Open text response
Provide 3 examples of what would help you better meet the needs of Veteran consumers	Open text response
CIL and VA collaborations	
Does your CIL have any formal collaboration (via contract) with the VA? (for example, housing, vocational rehab, peer support, etc.). Please specify type:	We have formal collaboration(s)—please specify; Open text response for “Please specify type”; N/A
Does your CIL have any informal collaboration (via personal contact or referrals) with the VA? (for example, housing, vocational rehab, peer support, etc.). Please specify	We have informal collaboration(s)—please specify; Open text response for “Please specify type”; N/A
If you did collaborate with the VA in the past and are no longer working with them, can you summarize why the collaboration no longer exists?	Open text response; N/A
Name 3 things you would like to improve in your collaboration with the VA	Open text response
Any additional comments	Open text response

## Survey Administration

The survey was administered via Qualtrics, a secure web-based survey platform. The web-based format enabled use of branching logic to gain greater insight into the different types of services provided by the CIL. Consistent with the voluntary nature of the survey, no items were selected for required response. The survey was open until June 2019, with the last surveys completed on June 3, 2019. Survey data were exported to a Microsoft Excel file from the web-based platform and maintained behind the research team’s secure VA firewall.

## Analysis

Data were first inspected for completeness and for the presence of duplicate internet protocol (IP) addresses. Responses that were completely blank were excluded from analysis. Responses with duplicate IP addresses, where responses were obtained for at least one item, were merged to create one representative respondent, which was then used in the analysis. For responses from duplicate IP addresses that contained any amount of data, all responses were compared and the following rules applied: (1) For continuous data, all responses provided were averaged with the average value used as the representative datapoint. (2) For categorical data, when values were identical, the categorical rating was retained. When the categorical ratings were discrepant, the representative datapoints were considered missing. (3) For textual data from open-ended survey questions, all data were retained and included in the qualitative analysis.

Descriptive statistics were calculated for quantitative data. For numerical data characterizing CIL respondents, additional variables were calculated to enhance dimensionality of the characterization. These include calculations establishing the percentage of CIL consumers that were Veterans and estimations of the CIL budgets relative to the number of consumers served. Qualitative data were independently examined by two members of the research team for patterns and categories in the textual data. Discrepancies were discussed among the coders and with other team members to arrive at consensus.

## Results

From the initial wave of survey recruitment, 383 email invitations were sent, of which 35 (9.1%) were undelivered and 56 (14.6%) responses were received. The response rate of the first wave was 16.1%, excluding 35 (9.1%) undeliverable emails. The second wave of 383 email invitations/reminders yielded 3 (< 1%) undeliverable emails and an additional 139 (36.3%) responses. The total response rate following the second invitation was 51.3% (*n* = 195), excluding the 3 undeliverable emails. The third and final wave of 383 email invitations/reminders yielded 4 (1.0%) undeliverable and an additional 23 responses, totaling 218 responses from the three waves of survey recruitment. The response rate of the final wave reached 57.5%, excluding the 4 undelivered emails.

From the 218 responses, 169 unique IP addresses were identified. Of these, 40 did not provide data (i.e., blank) and were excluded from analysis resulting in 129 unique IP addresses for analysis and a final response rate of 33.7%. The final sample (*N* = 129) included 36 responses from16 unique IP addresses, which were merged into 16 representative responses. The final sample comprised CILs from 39 states (Table 2). The following regions had states in which no CILs responded: South (Alabama), Northeast (Maine, New Hampshire, Vermont), Midwest (Nebraska, Oklahoma), and West (Montana, Nevada, South Dakota, and Wyoming).

**Table 2:** Respondents (*N* = 129) by state to survey of Centers of Independent Living (CILs)

State	CIL respondents (*n*)	CIL respondents tracking Veteran consumers (*n*, [% by state])	Missing/no response (*n*)
Alaska	3	1 (33.3)	0
Arizona	3	2 (66.7)	0
Arkansas	1	1 (100)	0
California	6	1 (16.7)	0
Colorado	6	2 (33.3)	2
Connecticut	1	1 (100)	0
Delaware	1	1 (100)	0
Florida	8	7 (87.5)	0
Georgia	3	3 (100)	0
Hawaii	1	1 (100)	0
Idaho	3	0 (0)	0
Illinois	6	2 (33.3)	0
Indiana	3	1 (33.3)	0
Iowa	3	2 (66.7)	0
Kansas	3	2 (66.7)	0
Kentucky	2	0 (0)	0
Louisiana	1	1 (100)	0
Maryland	2	2 (100)	0
Massachusetts	1	0 (0)	0
Michigan	6	2 (33.3)	1
Minnesota	4	3 (75)	0
Mississippi	1	0 (0)	0
Missouri	12	5 (41.7)	1
New Jersey	2	0 (0)	0
New Mexico	1	0 (0)	1
New York	11	7 (63.6)	0
North Carolina	1	0 (0)	0
North Dakota	2	2 (100)	0
Ohio	4	2 (50)	0
Oregon	1	1 (100)	0
Pennsylvania	5	3 (60)	0
South Carolina	2	1 (50)	0
Tennessee	1	0 (0)	0
Texas	3	2 (66.7)	1
Utah	3	2 (66.7)	0
Virginia	8	2 (25)	1
Washington	1	1 (100)	0
West Virginia	2	1 (50)	1
Wisconsin	2	2 (100)	0

## CIL Characteristics

Almost half the CILs had ≥ 26 staff (Table 3). CILs reporting the least staff (1–5 members) were located in Arizona, Arkansas, Delaware, Florida, Georgia, Iowa, Missouri, Ohio, South Carolina, and Virginia. CILs with the largest number of staff (26 or more) were located in Alaska, Arizona, California, Colorado, Florida, Georgia, Idaho, Illinois, Kansas, Kentucky, Louisiana, Massachusetts, Michigan, Minnesota, Mississippi, Missouri, New Jersey, New Mexico, New York, Ohio, Pennsylvania, South Carolina, Texas, and Wisconsin. The CIL reporting the largest operating budget and the one reporting the lowest operating budget were both in Arizona.

**Table 3:** Characteristics of Centers for Independent Living (CILs) (*N* = 129)

Characteristic	Descriptive statistic
Number of staff members	
≤ 5 staff	12* (9.5%^†^)
6–15 staff	43 (34.1%^†^)
16–25 staff	24 (19.0%^†^)
≥ 26 staff	47 (37.3%^†^)
Missing	3 (2.3%**)
Operating budget in fiscal year 2018	
Mean	$2,738,769.61
Standard deviation	$2,801,458.61
Median	$1,000,000.00
Quartile 1	$575,000.00
Quartile 3	$2,200,000.00
Interquartile range	$1,625,000.00
Missing	24* (18.6%**)
Number of consumers served in fiscal year 2018	
Mean	2134.6 consumers
Standard deviation	2094.5
Median	1000
Quartile 1	400
Quartile 3	1764
Interquartile range	1364
Missing	4* (3%**)
Percentage of CIL consumers in fiscal year 2018 who were rural	
Mean	49.1%
Standard deviation	33.2
Median	38.5
Quartile 1	15
Quartile 3	94.1
Interquartile range	79.1
Missing	11* (9%**)
Percentage of CIL consumers in fiscal year 2018 who were Veterans	
Mean	7.2%
Standard deviation	5.9
Median	4.6
Quartile 1	1.7
Quartile 3	9.6
Interquartile range	7.9
Did not track Veterans	55* (42.6%**)
Missing	8* (6.2%**)
Annual budget relative to number of CIL consumer^α^	
Mean	$2,227.02 per consumer
Standard deviation	$1,758.04
Median	$1,313.91
Quartile 1	$615.77
Quartile 3	$1,313.91
Interquartile range	$2,133.11
Missing	25* (19%**)

*Count

**Percentage of the entire sample (*N* = 129)

^†^Percentage of the sample that reported on the item

^α^Ratio of dollars per consumer as calculated by dividing respondent’s reported budget by reported number of CIL consumers

The five CILs reporting the highest number of consumers in fiscal year 2018 were from the following states: New York (52,000 consumers), Arizona (20,221 consumers), Michigan (13,850 consumers), Pennsylvania (12,000 consumers) and California (10,000 consumers). The five CILs reporting the lowest number of consumers were from California (48 consumers), Illinois (112 consumers), Kentucky (199 consumers), and Iowa (120 consumers). A total of 27 CILs (20.9%) reported serving 100% rural consumers. These CILs were located in the following states (when more than one CIL in the state reported a fully rural consumer base, the number of CILs reporting the rural consumer base is indicated in parentheses): Alaska, Arizona, Colorado, Georgia, Hawaii, Idaho, Illinois, Kansas, Michigan (2), Minnesota, Mississippi, Missouri (6), New York (4), Oregon, Tennessee, Utah, and Virginia (2). Only four CILs (3.1%) reported serving entirely urban consumers and were located in the following states (with the number of multiple CILs from the same state that are serving no rural consumers indicated in parenthesis): California, Missouri, New York (2).

Approximately half (*n* = 66; 51.2%) of CILs reported tracking Veteran status of consumers. Of these, the CIL with the largest percentage of Veteran consumers was in Georgia, whose consumer population was 44.4% Veteran. Nine CILs from the following 8 states reported Veteran populations of less than 1%: Colorado, Delaware, Missouri, New York (2 CILs reporting), Ohio, South Carolina, and Virginia.

Calculations of reported annual budget relative to number of consumers served yielded the highest ratios for three CILs in the following states: Pennsylvania, with a ratio of $19,167.00 per consumer; Colorado with a ratio of $11,143.00 per consumer; and California with a ratio of $10,417.00 per consumer. Three CILs from the following states had the lowest annual budget/consumer: Pennsylvania, $29.00/consumer; New York, $135.00/consumer; and Michigan, $152.00/consumer.

## CIL Services Used by Veteran Consumers

**Fig. 1:**
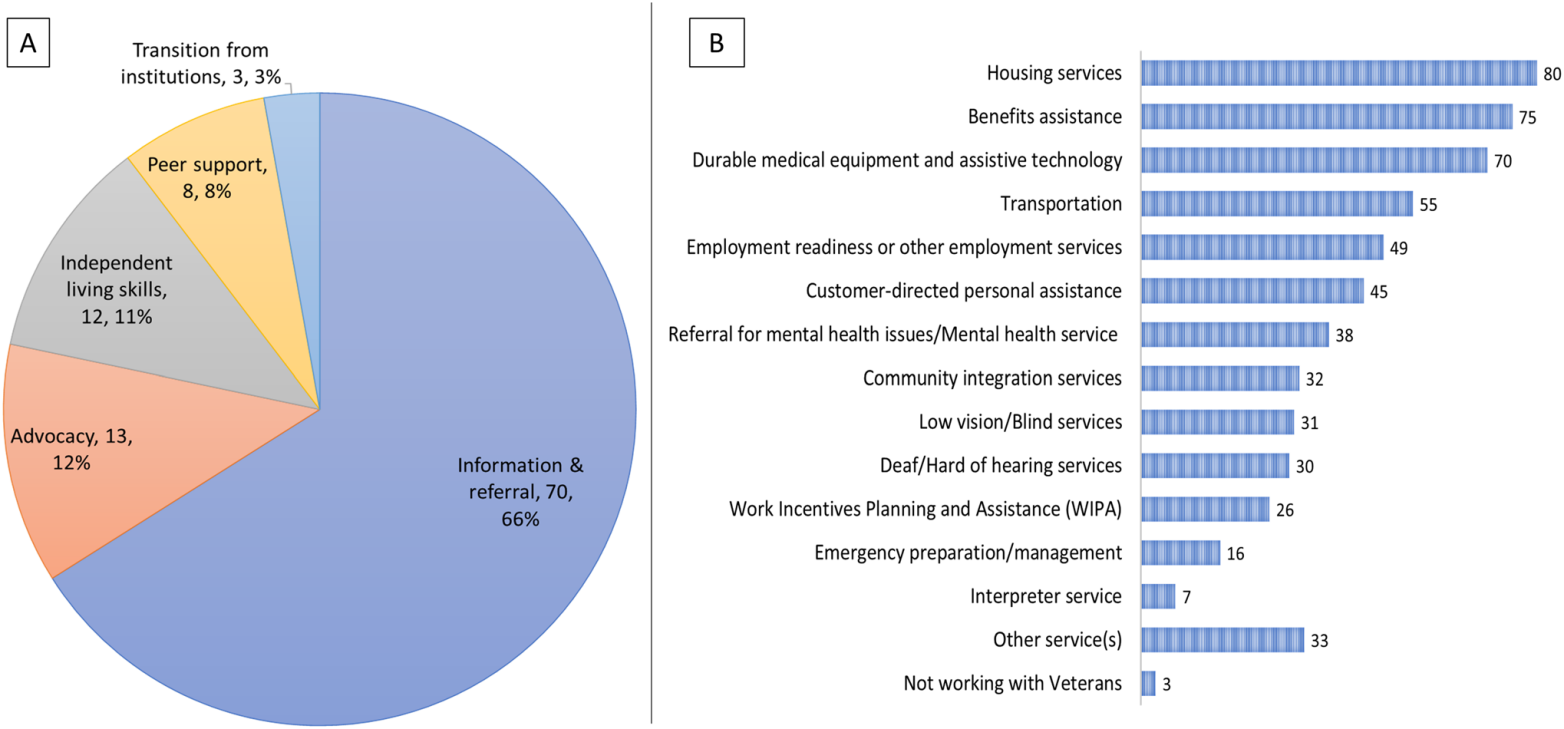
Centers for Independent Living (CIL) services used by Veterans **a** Details the service identified as the most frequently used from among the five core services (information and referral, independent living skills, peer support, advocacy services and transition assistance) as reported by 106 CILs. **b** Details frequency of CIL services (other than the five core services) that were identified as frequently used by Veteran consumers as reported by 120 CILs

One hundred twenty CIL respondents (93%) reported on services most used by their consumers who are Veterans. The services most frequently used by CIL Veteran consumers included services for information and referral, housing-related supports, assistance with understanding and navigating healthcare and Veterans benefits, supports for obtaining or using needed durable medical equipment and assistive technologies, and supports for meeting transportation and community mobility needs (Fig. 1).

**Fig. 2:**
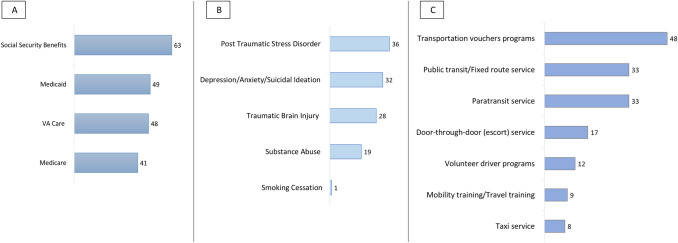
Types of benefits assistance, mental health referral and transportation services used by Veteran consumers at Centers for Independent Living (CILs) **a** Types of benefits assistance services used by Veteran consumers as reported by 72 CILs. **b** Types of referrals for mental health issues/mental health services used by Veteran consumers as reported by 37 CILs. **c** Types of transportation services used by Veteran consumers as reported by 55 CILs. *Note:* 75 of the 129 survey respondents who identified transportation as a frequently used service were queried via survey branching as to specific types of benefits assistance services used by Veteran consumers; 38 of the 129 survey respondents who identified mental health referral as a frequently used service were queried as to specific types of referral for mental health issues/mental health services; and 55 of the 129 survey respondents who identified transportation supports as a frequently used support were queried as to the specific types of transportation services used by Veteran consumers

Over half of CILs (*n* = 75; 58.1%) reported that their Veteran consumers were receiving CIL supports related to health and care benefits, of which 72 (96.0% of item respondents) responded to the follow-up survey item that queried as to specific the types of benefits supports provided. Supports related to Veteran’s Social Security benefits were the most frequently provided support related to benefits (*n* = 63; 87.5% of follow-up item respondents). Two-thirds of CILs providing supports related to benefits (*n* = 48; 66.7% of follow-up item respondents) reported providing their Veteran consumers supports related to VA health services (VA care). Fewer CILs reported providing Veteran consumers supports related to Medicaid (*n* = 49; 68.0% of follow-up item respondents) or Medicare (*n* = 41; 56.9% of follow-up item respondents) (Fig. 2a).

Almost one-third of CILs (*n* = 38; 29.5%) reported providing referrals related to mental health issues, of which 37 (97.3% of item respondents) responded to the follow-up survey item querying as to specific types of mental health issues. The most frequently reported referrals for mental health issues were reported for PTSD (*n* = 36; 97.3% of follow-up item respondents), followed by referrals related to depression, anxiety, and/or suicidal ideation (*n* = 32; 86.5% of follow-up item respondents) (Fig. 2b).

Almost half of CILs (*n* = 55; 42.6%) reported providing transportation supports or programs for Veteran consumers, of which all specified the types of transportation supports used by their Veteran consumers. The transportation programs most commonly provided by CILs and used by Veterans were transportation voucher programs (*n* = 48; 87.3% of item respondents), followed by public transit (*n* = 33; 60.0% of item respondents), and paratransit services (*n* = 33; 60.0% of item respondents) (Fig. 2c).

Thirty-three respondents (25.6%) provided open-ended text responses to the item “What other services are most often used by Veteran consumers at your CIL? (check all that apply)” when prompted by their selection of the “Others (please specify)” response. These respondents specified additional services used by Veteran consumers at their CILs that included home modifications, sports and recreation (including outdoor recreation and hunting), Veteran retreats, independent living skills training, shelter location for homeless Veterans, men's suit closet, transition from nursing facilities, and service dog programs for Veterans with PTSD.

## CIL/VA Collaborations

When queried as to existing relationships with the VA, 38 (29.5%) of CILs indicated the presence of formal (i.e., contracted) collaborations. Of the 38, 33 (86.8% of item respondents) detailed the nature of the formal collaboration(s) via open text response. Formal collaborations most often comprised the Veteran-Directed Care program, but also included a Memorandum of Understanding with the VA to offer access to a sports and fitness center for Veterans, the Long-term Care Ombudsprogram for VA nursing homes, and a contract with a state housing authority to work with Veterans.

A little more than half (*n* = 71; 55.0%) indicated that their CILs had informal collaborations with the VA, with 67 (94.4% of item respondents) describing the nature of their informal collaborations. Types of informal collaborations included contacts with counselors, social workers, and other VA staff for referrals on home modifications and durable medical equipment for Veterans; helping Veterans transition from nursing homes back to their own home; and problem solving with VA to help Veterans obtain benefits and employment.

Almost a quarter of the sample (*n* = 30; 23.3%) reported past but discontinued collaborations with the VA, with 28 (93.3% of item respondents) describing the nature of discontinued collaborations. Challenges to maintaining collaborations included a lack of CIL understanding of VA benefits available for Veterans and a perceived lack of VA understanding of CILs′ independent living philosophy.

## Specific Needs of Rural Veteran Consumers

In response to the open-ended question about the greatest needs of rural Veterans, 104 respondents (80.6%) indicated needs that included “*transportation*” or “*rural transportation*”*, "housing",* specifically “*affordable and accessible housing*”, “*employment*” and “*employment supports*”, “*peer support*”, “*mental health care*”, “*personal care attendants*”, “*home modifications*”, “*community integration*”, “*assistive technology*”, “*medical care*”, “*substance use services*”, “*independent living skills*”, “*financial supports*” and “*basic income*”.

One respondent explained that rural Veteran needs were “*all over the board*,” but that, in sum, these needs predominantly revolved around “*how to use community-based services to remain at home*”. Another respondent emphasized what they perceived as a dire need for housing by filling in the open-ended text box with: “*Housing, housing, housing–especially accessible AND affordable housing*”. A majority of respondents also reported transportation as one of the greatest needs, one respondent specifying that “*rural Veterans need better VA transportation options to clinics and resource Centers*”, including “*access to VA clinics*”. Reporting from Hawaii, a place with substantial rural transportation issues due to island geography, a respondent wrote: “*This state is made up of several islands. The Big Island of Hawaii is the only island that has a VA Center where Vets can get information on benefits they may qualify for and find a safe place to talk to like kin. Please remember no one can drive to Oahu if they live on another island*.”

Notably, many respondents underscored the need for more information about access to benefits for rural Veterans. This included “*disability benefits*”, “*VA benefits*”, and desiring an “*understanding [of] benefits*” in general, with “*knowledge of what is available within and outside the VA system*”. One respondent asserted the importance of “*meeting one-on-one, face to face with a person who can assist them through all the [benefits] paperwork and processes; someone who knows the services and someone willing to go to the Veteran, not make the Veteran travel.*” Another respondent specified the need to provide “*assistance with understanding disability rights laws and financial supports*”, while another explained that their Veteran consumers needed more information about eligibility for Veteran benefits: “*We see a majority of our consumers who are veterans that have a lack of knowledge of the VA benefits they are eligible for*”.

Mental health support was also frequently reported as one of the greatest needs of rural Veterans. One respondent wrote that investment in support groups for mental healing specifically through the kind of peer approach promoted by CILs would be a way to overcome the stigma of mental health issues: “*More funding for support groups and campaigns to stamp out the stigma of mental illness through a peer approach is needed in Veteran Services…such as support groups.*” Another respondent suggested that options to provide mental and social support beyond the VA are needed, writing of “*the need to form healthy social alliances and non-government options for healing*”.

## What is Needed to Better Meet the Needs of Veteran Consumers

When asked to provide textual examples of what would help CILs better meet the needs of Veterans, 109 respondents (84.5%) provided at least one example. The examples provided were consistent with what was reported as the greatest needs of rural Veterans: transportation services and housing supports. One respondent reported, “*We do not charge for our transportation services so we are always looking for more [transportation] funding*.” Other respondents independently reported that “*more accessible transportation options for travel to medical services*” and “*increased availability of intercounty medical transportation*” would help Veterans. Also mentioned by different respondents were “*transportation that goes [from rural areas] to services in urban areas or bring(s) services to rural areas*”, “*transportation vouchers to points of service*”, “*longer operating hours for transportation [supports]*”, and “*additional transit funding*”.

In addition, respondents reported wanting to find ways to better help Veterans with “*homelessness and mental health issues*” and to help Veterans find "*affordable housing*" and “*housing services*”. One respondent noted that policy change was needed to “*approve low income accessible housing projects*”. Others responded that what could help them better meet the needs of Veterans was “*targeted access to home modification funding*” and more “*home modifications for accessibility*”. Related goals for supporting Veterans’ independent living at home included “*preventing [Veterans] from going into nursing homes*”, “*self-directed in-home personal care*”, “*access to in-home support*”, and “*funding for individualized home care needs and community living supports*”. On the topic of in-home supports, another respondent promoted raising compensation rates and providing more adequate funding for attendants and in-home aids, one “*that supports paying a reasonable wage and not near minimum wage*”. Other frequently reported examples of what could better help to meet the needs of Veteran consumers revolved around increasing social and mental health support to Veterans; these included “*creative programs*”, “*service dog programs*”, and “*veteran peer support programs*”.

Beyond accessible and affordable housing, transportation, and social support to Veterans, respondents most frequently suggested that improving collaboration with the VA and other Veteran service organizations is what is most needed to better meet the needs of Veterans. Many emphasized a need for better and direct communication with the VA, the ability to “*work more closely with local veteran services office*” and “*collaborative meetings with VA representatives and social workers*”. Multiple respondents also independently reported a desire for “*more education on benefits available to VA enrollees*”, “*more familiarity with the services [Veterans] may qualify for*”, knowledge of “*the rules so we could help make sure [Veteran’s benefits/services] happens*” and “*training for CIL staff on Veterans Benefits and how to access them*”. Respondents also reported that a greater “*understanding of the disability rating system for Veterans and what services they can access within a civilian service agency*” would help CILs to better help Veterans, as would “*more consistent information (whether by e-mail, phone contact or postal service) regarding services available for disabled veterans*”. Respondents also reported perceiving that the VA and other Veteran service organizations lack awareness of what CILs can offer to Veteran consumers. They called for both “*Veteran services to... develop a white paper on how CIL services may benefit Veterans*” and for CILs to provide greater “*outreach to veterans*” through “*dedicated staff*”.

## Discussion

This study examined the perspectives of CIL directors regarding the needs of Veteran consumers, especially rural Veterans, and how CILs are currently serving them. This national survey of CILs obtained the necessary data to begin understanding how long-standing, non-VA community-based resources such as CILs are enhancing independent living among rural Veterans and what potential avenues exist for further collaboration. The national representation of CILs among respondents is a strength of the present study, providing a picture of the broad extent to which CILs are especially attuned to rural Veteran needs for services and supports. Importantly, despite the broad range of respondents’ CIL size, as well as budget, and variation in whether CILs are tracking the military status of their consumers, we found consistency and overlap in CILs′ understanding of rural Veteran needs. Notably, there was also substantial consistency in the perception of what CILs need overall in order to better support their Veteran consumers.

While on average just over 7% of CIL consumers were reported to be Veterans, we anticipate that this number represents an underestimation of Veterans actually served by CILs. Almost half of CILs reported not tracking Veteran status. In addition, for those CILs that do track Veterans, Veterans may not disclose that they served in the military if they received a less than honorable discharge. They also may not self-identify as Veterans if they were not career or combat military, or alternatively, if they do not meet the VA’s eligibility criteria for benefits. It is also unknown whether CILs who reported tracking Veterans are specifically asking at intake whether an individual consumer has “ever served in the military,” which may yield more accurate data. It has been recommended that civilian care providers ask during intake “Have you ever served in the military?” [25].

Veteran consumers of CILs access information and referral (including on healthcare navigation and benefits counseling), housing-related supports, durable medical equipment and assistive technologies, and transportation and community mobility services through CILs. CIL data revealed that CILs most often provide benefits counseling related to Social Security benefits and secondly to VA benefits, but they also provide supports related to Medicare and Medicaid. This finding indicates that Veterans are accessing multiple types of benefits and that they are relying on CILs to help them navigate the complicated benefits systems. Rural Veterans have been reported to be especially unaware of VHA healthcare benefits [26] and may need support navigating their options of services or care. Importantly, prior published studies have found that improving rural Veterans’ knowledge of and access to dual care services predicted satisfaction with their healthcare [27], therefore CILs who support Veterans in navigating multiple streams of care and benefits may likely play a role in increasing Veterans′ overall satisfaction.

That respondents identified homelessness and housing as some of the greatest challenges for their Veterans is consistent with the literature citing the overlap of less than honorable discharge for Veterans being associated with higher risk of housing instability [28] as well as mental health challenges being associated with unstable housing [29]. Moreover, respondents overwhelmingly referred to a need for home modification support. Recent research shows that VHA programs—such as HISA that provides home repair to enable Veterans with special needs to return to their homes after hospitalization—or Veterans Benefits Administration programs that facilitate home modifications, may be underutilized by Veterans in the community [30].

In terms of transportation needs, while many VAs provide transportation programs for Veterans enrolled in tailored programs for homeless Veterans [31], CILs may be an important transportation resource for Veterans who are not enrolled in these programs. This is especially noteworthy as literature has demonstrated that transportation is key for Veteran outcomes, for example, in their success with employment programs [32]. CILs also provide important social support for their Veteran consumers, an aspect of health and well-being that has been shown to be significantly related to better outcomes for at-risk Veterans [33]. Moreover, at least a third of respondents reported that CIL Veteran consumers commonly need supports for PTSD, depression, anxiety, and/or suicidal ideation. Challenges related to social isolation, which can undermine psychological recovery, can be exacerbated by rurality [34].

By virtue of both their philosophical origins and broad catchment areas, CILs are willing and well positioned to assist in closing the service and resource gaps that rural Veterans with disabilities face. CIL staff contribute to the well-being of their consumers through their own lived experience with disabilities themselves [35]. As such, they offer Veterans with disabilities authentic peer support and practical guidance in navigating the challenges of living with a disability. CILs employees can share their personal histories as inspiration for what is possible, which can help with Veterans with disabilities maintain a positive outlook and effectively manage their independent living needs.

CILs recently received funding through the CARES Act to expand their capacity to provide services to disabilities virtually [36]. Most CILs are now equipped with electronic platforms and devices that allow them to deliver services remotely. This expanded capacity is especially important for Veterans living in rural areas because it eliminates transportation barriers. Virtual platforms also allow consumers with disabilities to participate in group discussions and support groups from their homes and may likely facilitate more frequent participation.

Overwhelmingly, respondents, through open text responses, demonstrated their willingness and desire to meet the multifaceted needs of their Veteran consumers. They also demonstrated their knowledge of, and desire to ameliorate, the particular challenges that Veterans living in rural communities face.

## Limitations and Future Research

While the focus of the study was to better understand how CILs serve rural Veterans with disabilities, the questions asked primarily allowed for broad characterizations of how CILs are serving Veterans as well as what the major needs are for rural Veterans specifically; the questions did not lend themselves to understanding more deeply the services provided by CILs specifically to Veterans residing in rural areas. As such, understanding as to the most salient CIL services currently provided for rural Veterans remains preliminary.

We noted that outliers existed in our dataset regarding CIL budgets and numbers of consumers served, and thus reported both mean and median measures of central tendency. We did not exclude the observed outlying datapoints because of the exploratory nature of this study, whereby we did not want to exclude data that may accurately reflect the experiences of any one of the CILs. However, because invitations for study participation were only extended to main CILs, outlying datapoints may be reflective of centers who may or may not have incorporated information of their associated satellite centers.

In addition, while the majority (97%) of the sample of main CILs reported serving at least some rural consumers, the inclusion of satellite CILs in the sampling frame could have provided even greater understanding of rural Veteran consumers’ needs. Future studies should seek to incorporate the perspectives and experiences of satellite CILs, as many satellite CILs are located in rural regions.

Moreover, while the value of CIL services is increasingly documented in the literature [22, 37, 38], how CILs can and do provide support to Veterans is an area that would benefit from much more research. Further study is warranted to address remaining gaps in understanding possibilities for leveraging CIL services in ways that can best meet the multifaceted and complex needs of rural Veterans with disabilities.

## Conclusion

This paper provides the first systematic examination at a national level of the ways in which CILs can and do support Veteran consumers, especially those living in rural communities. In total, results from our study indicate that CILs may be providing an important safety net for Veterans with disabilities. While the VA works to address the needs of rural Veterans, there are still many rural Veterans who are not enrolled in the VA system or who may rely on CILs and other community resources to support their independent living needs. There is an urgent need for those who serve Veterans to better understand community-based resources such as CILs, and their services, history, and philosophy. As such, there exists a critical need for enhancing collaborations between CILs and local VA in order to achieve the goal of better meeting the needs of Veterans.

## Data Availability

The datasets generated during and/or analyzed during the current study are not publicly available, as per the Department of Veterans Affairs Veterans Health Administration Handbook 1200.12.
